# Current provision for MRI scanning of patients with cardiac implantable electronic devices - a national survey of hospitals in England

**DOI:** 10.1186/1532-429X-18-S1-O125

**Published:** 2016-01-27

**Authors:** James L Oldman, Kian Sabzevari, Anna S Herrey, James Moon, Anna C Kydd, Charlotte Manisty

**Affiliations:** 1 Department of Cardiac Imaging, Barts Heart Centre, London, UK; 2 grid.83440.3b0000000121901201Institute of Cardiovascular Science, University College London, London, UK

## Background

With increasing numbers of patients requiring implantation of a cardiac implantable electronic device (pacemaker or defibrillator), and MRI becoming the gold standard investigation for the diagnosis and monitoring of many medical conditions, device manufacturers have developed MRI conditional CIEDs. Many hospitals now implant MRI conditional devices as standard, meaning that there are large numbers of patients with these devices who believe that accessing and undergoing MRI scans with their devices should be straightforward. Unfortunately the reality is often very different; patients anecdotally report extreme difficulties with many MRI units refusing to scan them.

We sought to establish current provision for MRI scanning of patients with CIEDs in England, and the potential barriers to service expansion.

## Methods

A survey was distributed to all hospitals in England with MRI, to assess current practice. Information requested included whether hospitals currently offer MRI to this patient group, the number and type of scans acquired, local safety considerations, complications experienced and perceived obstacles to service provision in those departments not currently offering it.

## Results

Responses were received from 195 of 227 (86%) of hospitals surveyed. Although 98% of departments were aware of MR-conditional devices, only 46% (n = 89) currently offer MRI to patients with CIED's; with only 1 in 7 of those centers scanning more than 10 patients a year. Only 4% (7 out of 195) of departments currently offer MRI scans to patients with non MR-conditional CIEDs in situ.

No major complications were reported from MRI in patients with MR-conditional devices. Current barriers to service expansion include perceived concerns regarding potential risk, lack of training, logistical difficulties and lack of cardiology support.

## Conclusions

Provision of MRI scanning for patients with CIEDs is currently poor in England, despite increasing numbers of patients with MR-conditional devices and extremely low reported complication rates. Cardiology and Radiology need to work together at a national level to break down current barriers so that all eligible patients can benefit from MRI.

**Table 1:** Complications reported by MRI departments scanning patients with cardiac implantable electronic devices

Reported complications from hospitals offering MRI scanning to patients with cardiac implantable electronic devices	N (%)
None	82 (92%)
Minor Complications (e.g. Device parameters altered and re-programming required)	5 (6%)
Serious Complications (e.g. Arrhythmias, pacemaker malfunction requiring replacement)	1* (1%)
Not specified	1 (1%)

* Reported complication in a patient with a non MR-conditional CIED

**Figure 1:**
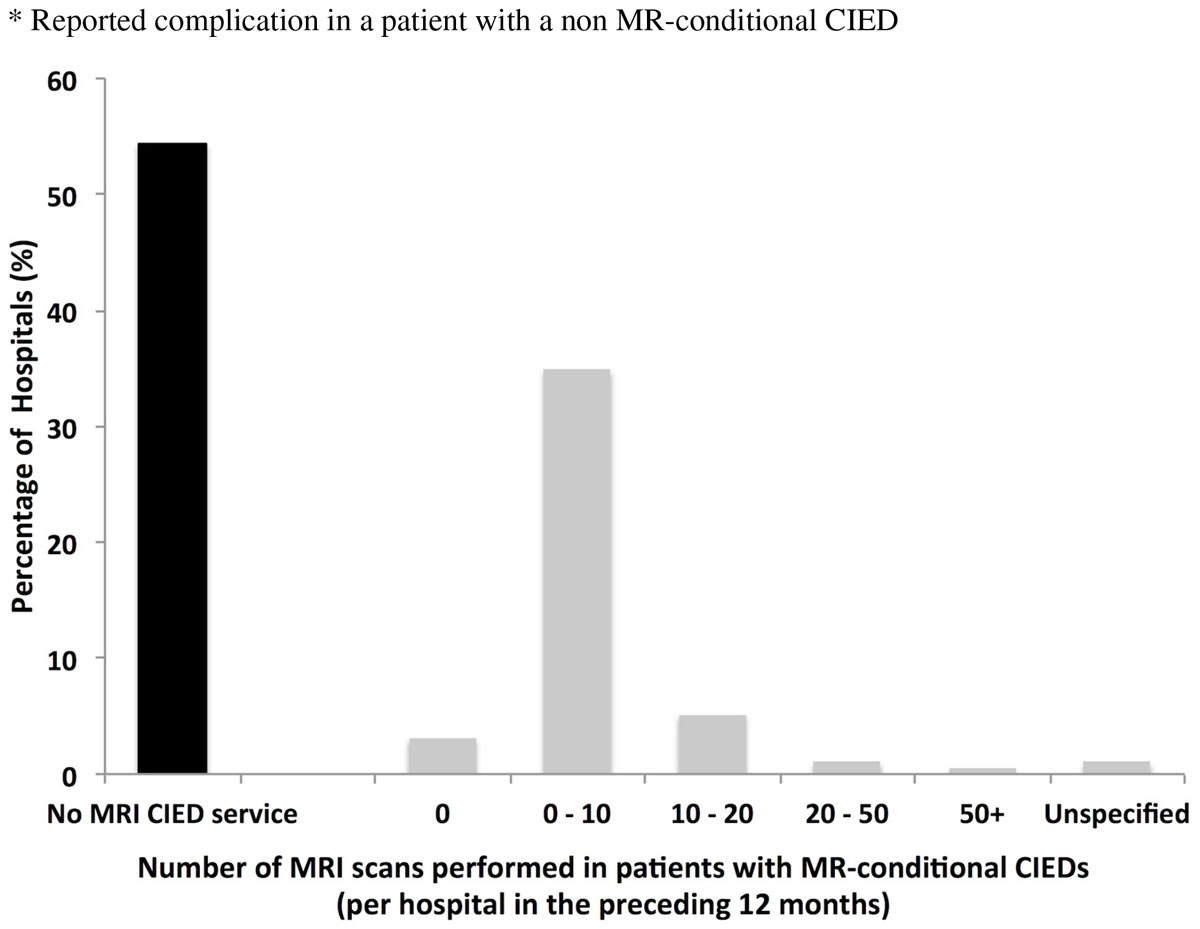
Figure 1

